# Birth outcomes in South African women receiving highly active antiretroviral therapy: a retrospective observational study

**DOI:** 10.1186/1758-2652-14-42

**Published:** 2011-08-15

**Authors:** Karin van der Merwe, Risa Hoffman, Vivian Black, Matthew Chersich, Ashraf Coovadia, Helen Rees

**Affiliations:** 1 Empilweni Services and Research Unit, Department of Paediatrics and Child Health, Rahima Moosa Mother and Child Hospital, University of the Witwatersrand Johannesburg, South Africa; 2 Wits Reproductive Health and HIV Institute, University of the Witwatersrand Johannesburg, South Africa; 3 David Geffen School of Medicine at UCLA, Division of Infectious Diseases, Los Angeles, California, USA; 4 Centre for Health Policy, School of Public Health, University of the Witwatersrand, Johannesburg, South Africa; 5 International Centre for Reproductive Health, Department of Obstetrics and Gynaecology, University of Gent, Belgium

## Abstract

**Background:**

Use of highly active antiretroviral therapy (HAART), a triple-drug combination, in HIV-infected pregnant women markedly reduces mother to child transmission of HIV and decreases maternal morbidity. However, there remains uncertainty about the effects of *in utero *exposure to HAART on foetal development.

**Methods:**

Our objectives were to investigate whether *in utero *exposure to HAART is associated with low birth weight and/or preterm birth in a population of South African women with advanced HIV disease. A retrospective observational study was performed on women with CD4 counts ≤250 cells/mm^3 ^attending antenatal antiretroviral clinics in Johannesburg between October 2004 and March 2007. Low birth weight (<2.5 kg) and preterm birth rates (<37 weeks) were compared between those exposed and unexposed to HAART during pregnancy. Effects of different HAART regimen and duration were assessed.

**Results:**

Among HAART-unexposed infants, 27% (60/224) were low birth weight compared with 23% (90/388) of early HAART-exposed (exposed <28 weeks gestation) and 19% (76/407) of late HAART-exposed (exposed ≥28 weeks) infants (p = 0.05). In the early HAART group, a higher CD4 cell count was protective against low birth weight (AOR 0.57 per 50 cells/mm^3 ^increase, 95% CI 0.45-0.71, p < 0.001) and preterm birth (AOR 0.68 per 50 cells/mm^3 ^increase, 95% CI 0.55-0.85, p = 0.001). HAART exposure was associated with an increased preterm birth rate (15%, or 138 of 946, versus 5%, or seven of 147, in unexposed infants, p = 0.001), with early nevirapine and efavirenz-based regimens having the strongest associations with preterm birth (AOR 5.4, 95% CI 2.1-13.7, p < 0.001, and AOR 5.6, 95% CI 2.1-15.2, p = 0.001, respectively).

**Conclusions:**

In this immunocompromised cohort, *in utero *HAART exposure was not associated with low birth weight. An association between NNRTI-based HAART and preterm birth was detected, but residual confounding is plausible. More advanced immunosuppression was a risk factor for low birth weight and preterm birth, highlighting the importance of earlier HAART initiation in women to optimize maternal health and improve infant outcomes.

## Background

In South Africa, hyper-pandemic levels of HIV persist, with few signs of a reduction in new HIV infections. Approximately one-third of women attending antenatal clinics in Gauteng Province are HIV infected [1]. Use of highly active antiretroviral therapy (HAART), a triple-drug combination, in HIV-infected pregnant women prevents mother to child transmission of HIV (MTCT) and reduces maternal morbidity [2,3] and mortality. However, there remains much uncertainty about whether *in utero *exposure to HAART affects foetal and later child development.

Many European studies have detected an association between protease inhibitor-based HAART and preterm birth [4,5], while the majority of North American studies have shown no such association [6-8]. Other evidence comparing birth outcomes in HIV-exposed infants before and since the introduction of HAART indicates that rates of low birth weight (LBW) and preterm birth have decreased since the introduction of HAART [9]. Studies to date have mostly been performed in high-income countries where HAART is initiated, regardless of maternal CD4 cell count and stage of HIV disease, for prevention of MTCT [4,6,10,11].

Moreover, in these studies, many women acquired HIV from intravenous drug use, and a large portion smoked during pregnancy, making it difficult to directly compare these populations with those in low- and middle-income countries [11]. In South Africa, prior to April 2010, pregnant women only initiated HAART with a CD4 count below 200 cells/mm^3 ^or an AIDS-defining illness. Further, clade C is the most common HIV strain in the country, and HIV is predominately acquired during heterosexual sex, with insignificant parenteral transmission.

There is limited evidence about HAART and birth outcomes in Africa. A study in Abidjan, Côte d'Ivoire, compared women eligible for HAART from two cohorts, each with approximately 150 women [12]. Low birth weight (<2.5 kg) was two-fold higher in the cohort that took three-drug HAART compared with the cohort that received two-drug, short-course antiretroviral prophylaxis for preventing MTCT. LBW rates were highest in those who had initiated HAART prior to pregnancy. A larger study in Botswana found that HAART-exposed infants were smaller for gestational age than unexposed infants [13]. Effects of specific HAART regimens were not explored. Further study of infant outcomes following HAART is warranted in African settings, particularly within routine clinical services at public sector ARV clinics.

We previously examined the effects of different HAART regimens and duration of therapy on risk for MTCT [14], and now assess associations between these factors and LBW and preterm birth.

## Methods

This study reports on a retrospective observational cohort of women attending integrated antenatal and antiretroviral (ANC-ARV) clinics at Rahima Moosa Mother and Child Hospital (RMH) and Charlotte Maxeke Johannesburg Academic Hospital (CMJH). Both clinics are referral centres for HIV-infected pregnant women in Johannesburg, but the latter provides care for patients with more complex medical conditions.

Women attending ANC-ARV clinics at the hospitals and all mother-infant pairs at the postnatal clinic of RMH were eligible for the study if they: were HIV positive; had a singleton delivery between October 2004 and March 2007; attended an ANC-ARV clinic during pregnancy or the postnatal clinic less than two months postpartum; and had a CD4 count of ≤250 cells/mm^3^. CD4 cell counts were taken before HAART initiation for the HAART-exposed group, and during pregnancy or within three weeks postpartum for the HAART-unexposed group. Women attending the postnatal clinic between three weeks and two months after childbirth were included if they had a CD4 count result from during pregnancy. Most of the comparison group (HAART-unexposed women) had not received antenatal HAART because they were identified as HIV positive during labour or in the postnatal period (within three weeks of childbirth).

Women gave consent for their data to be included in the clinic database and used for research purposes. The study protocol was approved by the Human Research Ethics Committee of the University of the Witwatersrand (protocol number M070438).

HIV status was determined with parallel rapid HIV tests (First Response HIV Card test 1-2.0 [Kachigam, Daman, India] and Pareekshak HIV Triline card test [Bangalore, Karnataka, India]). CD4 cell count was measured using a Beckman Coulter Epics XL MCL cytometer (Fullerton, CA, United States) or Beckman Coulter TQ PREP (Fullerton, CA, United States). HIV infection status was diagnosed in infants of more than six weeks of age with a DNA polymerase chain reaction (PCR) (Amplicor HIV-1 DNA PCR version 1.5 assay; Roche Diagnostics, Inc., Alameda, CA, United States).

At RMH, most pregnant women eligible for HAART initiated lopinavir/ritonavir, stavudine and lamivudine. First-line therapy at CMJH consisted of nevirapine, stavudine, and lamivudine. In both hospitals, efavirenz was initiated after the first trimester for women taking concomitant tuberculosis (TB) treatment. Women presenting in the first trimester of pregnancy who were receiving efavirenz-containing regimens were changed to lopinavir/ritonavir, while women beyond the first trimester continued efavirenz-based therapy. The HAART regimen was categorized by first regimen used in pregnancy.

The primary aim of our study was to investigate the association between LBW, and HAART duration and regimen during pregnancy. LBW was defined as a birth weight below 2.5 kg and very low birth weight (VLBW) as less than 1.5 kg. For the study, early HAART was defined as initiation before 28 weeks of pregnancy and late HAART as initiation at ≥28 weeks of pregnancy. A secondary aim of the study was to examine associations between preterm birth and HAART exposure, with similar analyses of regimen type and duration. Preterm birth was defined as birth before 37 completed weeks of pregnancy and extremely preterm birth was defined at birth before 34 weeks of pregnancy.

Gestational age was determined by a clinician at the first antenatal visit, using a combination of ultrasound (when available), last menstrual period and symphyseal-fundal height. In women who did not attend antenatal care, gestational age was estimated by examining the infant at birth; this estimate was recorded on the infant's clinic card. Small for gestational age was defined as a birth weight below the 10^th ^percentile of expected weight for gestational age, birth weights above the 90^th ^percentile as large for gestational age, and those between the 10^th ^and 90^th ^percentiles as appropriate for gestational age [15].

### Statistical methods

Single data entry was done in Microsoft Access and analysis performed with Intercooled Stata 8.0 (Stata Corporation, College Station, TX, United States). Characteristics of HAART-exposed and unexposed women were compared using chi-square tests for categorical exposure variables and Student's t-test for continuous variables. As we were interested in assessing the effects of drug regimen on LBW independent of the role of infant HIV infection, we initially restricted multivariate analysis of LBW to HIV-negative infants and thereafter included all infants in a similar analysis. A similar multivariate analysis was performed for variables associated with preterm birth in which we included all infants with a known gestation at birth, regardless of HIV status.

Variables associated with LBW or preterm birth in univariate analysis (p < 0.1) were included in the initial multivariate model and retained if their removal markedly altered the model fit. Model fit was assessed using the likelihood ratio test and judgement about the size of changes to the model with and without the variable [16]. The model analyzed the odds of LBW or preterm birth according to regimen type: lopinavir/ritonavir-based, nevirapine-based and efavirenz-based HAART; CD4 cell categories (cells/mm^3^) of 0-49, 50-99, 100-149, 150-199 and 200-250; maternal age; anaemia (haemoglobin <11 g/dl); hypertension (systolic blood pressure [BP] >160 mmHg or diastolic BP >90 mmHg on two occasions four hours apart, or one diastolic BP >110 mmHg); and infant HIV status. Women in the early and late HAART groups were analyzed in separate multivariate models due to the presence of interaction: associations between the exposures (drug regimen) and outcome LBW and prematurity) varied according to the duration of HAART.

## Results

### Maternal characteristics in HAART-unexposed and exposed women

**Figure 1:**
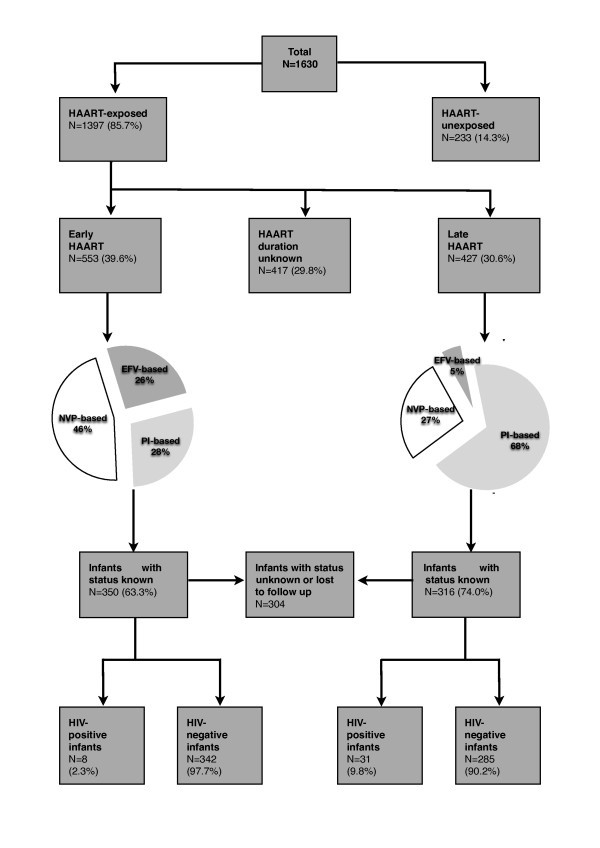
**Flow diagram of study population**. HAART - highly active antiretroviral treatment; early HAART was defined as initiation before 28 weeks of pregnancy and late HAART as initiation at ≥28 weeks of pregnancy; PI-based HAART - protease inhibitor-based HAART; EFV-based HAART - efavirenz-based HAART; NVP-based HAART - nevirapine-based HAART.

The cohort comprised 1630 women, with a mean age of 30.1 years (sd 5.1) and median CD4 count of 159 cells/mm^3 ^(IQR 105-200). Women who declined study participation were not included in the data base. Fourteen percent of included women (n = 233) were HAART unexposed. In the remainder (n = 1397) who received HAART during pregnancy, duration of therapy was known for 70%, with 54.5% of these (n = 533) initiating HAART before 28 weeks ("early HAART") and 427 at ≥ 28 weeks ("late HAART"; Figure 1).

Compared with unexposed women (Table 1), HAART-exposed women were older (30.3 vs. 28.9, p < 0.001), had a lower median CD4 count (154 cells/mm^3 ^vs. 191 cells/mm^3^, p < 0.001), and a lower rate of anaemia (46% [416/912] vs. 57% [57/100], p = 0.03). However, no differences were detected between these groups for other risk factors for LBW or preterm birth, such as gravidity, hypertension, alcohol use, smoking, syphilis and previous miscarriage. Of note, diabetes requiring treatment with medication (oral hypoglycaemics or insulin) was uncommon, with only four women in the cohort meeting these criteria. Positive syphilis serology (rapid plasma reagin) was detected in 3.8% (38/1001) of women.

**Table 1:** Demographics and maternal health status in women exposed and unexposed to antiretroviral treatment and by duration of exposure

Variable category	Variables	HAART-unexposed	HAART-exposed	P	Early HAART-exposed	Late HAART-exposed	P
		N = 233	N = 1397		N = 553	N = 427	
**Demographics**	**Maternal age**	N = 180	N = 1372		N = 540	N = 426	
	mean years (SD)	28.9 (5.1)	30.3 (5.1)	<0.001	30.6 (5.1)	30.1 (5.2)	0.18

**Substance use**	**Smoked in pregnancy**	N = 140	N = 777		N = 314	N = 312	
	n (%)	6 (4)	28 (4)	0.69	15 (5)	7 (2)	0.085
	
	**Alcohol use in pregnancy**	N = 144	N = 782		N = 317	N = 315	
	n (%)	7 (5)	29 (4)	0.51	15 (5)	7 (2)	0.085

**Immune status**	**CD4 count **cells/mm^3 ^n (%)	N = 233	N = 1397		N = 553	N = 427	
	0-49	5 (2)	124 (9)		59 (11)	28 (7)	
	50-99	29 (12)	216 (15)		91 (16)	62 (15)	
	100-149	35 (15)	325 (23)		122 (22)	108 (25)	
	150-199	64 (27)	412 (29)		156 (28)	136 (32)	
	200-250	100 (43)	320 (23)	<0.001	125 (23)	93 (22)	0.12
	median CD4 cell count	191	154		152	155	
	IQR	136-220	101-195	<0.001	93-195	108-194	0.12

**Health status**	Hypertension*	N = 145	N = 928		N = 410	N = 431	
	n (%)	18 (12)	93 (10)	0.38	41 (10)	27 (8)	0.32
	
	Diabetes**	N = 147	N = 932		N = 410	N = 344	
	n (%)	1 (1)	3 (0)	0.51	1 (0)	2 (1)	0.46
	
	**Haemoglobin **(Hb)	N = 100	N = 912		N = 312	N = 286	
	Hb <11 gm/dl, n (%)	57 (57)	416 (46)	0.03	129 (41)	140 (49)	0.062
	Median Hb gm/dl	10.7	11.1		11.3	11.0	
	IQR	9.8-11.5	9.9 -11.9	0.019	10.1-12.1	9.9-11.8	0.007
	
	**Syphilis serology **n (%)	N = 137	N = 864		N = 273	N = 339	
	positive RPR	3 (2)	35 (4)	0.29	11 (4)	11 (3)	0.60

**Reproductive health**	**Gravidity **n (%)	N = 204	N = 954		N = 413	N = 354	
	1	30 (15)	126 (13)		51 (12)	57 (16)	
	2	83 (41)	351 (37)		142 (34)	140 (40)	
	3	59 (29)	292 (31)		131 (32)	98 (28)	
	≥4	32 (16)	185 (19)	0.50	89 (22)	59 (17)	0.083
	median	2	2		3	2	
	IQR	2-3	2-3	0.13	2-3	2-3	0.010
	
	Previous miscarriage***	N = 146	N = 766		N = 313	N = 310	
	n (%)	27 (18)	130 (17)	0.66	70 (22)	38 (12)	0.001
	
	Previous preterm infant***	N = 148	N = 621		N = 231	N = 281	
	n (%)	16 (11)	39 (6)	0.055	13 (6)	18 (6)	0.71

All women have a CD4 ≤250 cells/mm^3^; HAART - highly active antiretroviral treatment; early HAART - HAART initiation <28 weeks of pregnancy; late HAART - HAART initiation at ≥28 weeks of pregnancy; SD - standard deviation; IQR - interquartile range; RPR - rapid plasma reagin * systolic blood pressure [BP] >160 mmHg or diastolic BP >90 mmHg on 2 occasions 4 hours apart; or 1 diastolic BP >110 mmHg

** Diabetes requiring medical intervention ***in women with a previous pregnancy

### Characteristics of women by clinic site and MTCT rates

Compared with women at RHM, those at CMJH were less frequently primigravidas (10% [49/487] vs. 16% at RHM [77/467], p = 0.005), more likely to have advanced WHO HIV disease (46% stage 1 [165/359] vs. 73% [281/385], p < 0.001) and more likely to have had previous miscarriages (22% [67/305] vs. 14% [63/450], p = 0.002). Information on TB was not available from RHM and was only available for 33% (466/1397) of women at CMJH, with 17% (80/466) of these having either current or prior TB.

A total of 1019 infants attended their scheduled visit for HIV DNA PCR testing at six weeks. Risks of MTCT were 3.6-fold higher in the non-HAART than HAART group (19% [37/191] versus 5% [45/828], p < 0.001), with the largest reduction in transmission among the early HAART group (2% [8/350] versus 10% [31/316] in the late group, p < 0.001).

### Maternal characteristics in early versus late HAART initiators

The median duration from HAART initiation to childbirth was 18.4 weeks (IQR 12.1-42.6) for the early HAART group and 5.8 weeks (IQR 3.3-8.5) for the late group (p < 0.001). Women in the early group more commonly received NVP-based HAART (46%, 254/553), while those in the late group were more likely to receive protease inhibitor-based HAART (68%, 290/427; Figure 1). No differences were detected between the early and late HAART groups in terms of baseline CD4 count, previous preterm birth rates, syphilis prevalence and smoking. However, women in the early HAART group had higher rates of previous miscarriage than the late HAART group (22% [70/313] vs.12% [38/310], p = 0.001).

### Infant outcomes: low birth weight

The mean birth weight for infants in the cohort was 2.88 kg, with 22.4% (275/1228) having LBW. Among infants with known HIV status, LBW was more common in HIV-positive than negative infants (33% [24/73] vs. 22% [173/804], p = 0.04). In HAART-unexposed infants, 27% (60/224) were LBW compared with 23% (90/388) of early HAART-exposed and 19% (76/407) of late HAART-exposed infants (p = 0.05). No differences, however, were detected when comparing rates of LBW in HAART-unexposed versus HAART-exposed infants (27% [60/224] vs. 21% [215/1004], p = 0.08), or between the early and late HAART groups (23% [90/388] vs. 19% [76/407], p = 0.12). Among women with early HAART-exposure, higher rates of LBW were observed in women receiving efavirenz-based regimens (38% [36/95]) compared with nevirapine (20% [31/158]) and protease inhibitor-based regimens (17%, [23/135]; p < 0.001). There were no differences in rates of LBW by regimen in the late HAART group (Table 2).

**Table 2:** Infant outcomes according to maternal antiretroviral regimens and duration of treatment in pregnancy

Variables	Early HAART-exposed			P	Late HAART-exposed			P
				
	PI-based HAART	NVP-based HAART	EFV-based HAART		PI-based HAART	NVP-based HAART	EFV-based HAART	
**Birth weight (kg): **n (%)	N = 135	N = 158	N = 95		N = 284	N = 103	N = 20	
mean (SD)	3.0 (0.6)	2.9 (0.5)	2.7 (0.6)	0.002	2.9 (0.5)	2.9 (0.5)	2.8 (0.5)	0.59
0.75-1.49	5 (4)	0 (0)	3 (3)		2 (1)	0 (0)	0 (0)	
1.5-2.49	18 (13)	31 (20)	33 (35)		46 (16)	23 (22)	5 (25)	
>2.5	112 (83)	127 (80)	59 (62)	<0.001	236 (83)	80 (78)	15 (75)	0.50

**Gestation: **n (%)	N = 131	N = 167	N = 91		N = 290	N = 116	N = 21	
extremely premature (<34 weeks)	13 (10)	15 (9)	12 (13)		0 (0)	3 (3)	0 (0)	
preterm (34-36 weeks)	6 (5)	25 (15)	10 (11)		9 (3)	8 (7)	1 (5)	
term/postdates (>36 weeks)	112 (86)	127 (76)	69 (76)	0.048	281 (97)	105 (91)	20 (95)	0.024

**Birth weight - gestation **n (%)	N = 123	N = 128	N = 61		N = 280	N = 101	N = 19	
AGA	83 (67)	110 (86)	39 (64)		153 (55)	79 (78)	13 (61)	
SGA	26 (21)	13 (10)	18 (30)		121 (43)	20 (20)	6 (32)	0.001
LGA	14 (11)	5 (4)	4 (7)	0.001	6 (2)	2 (2)	0 (0)	

**Infant HIV status**: n (%)	N = 106	N = 156	N = 88		N = 214	N = 87	N = 15	
HIV PCR positive	1 (1)	6 (4)	1 (1)	0.22	17 (8)	13 (15)	1 (7)	0.17

HAART - highly active antiretroviral treatment; early HAART - HAART initiation <28 weeks of pregnancy; late HAART - HAART initiation at ≥28 weeks of pregnancy; PI - protease inhibitor, NVP - nevirapine; EFV - efavirenz; SD - standard deviation; SGA - small for gestational age; AGA - appropriate for gestational age; LGA - large for gestational age; PCR - polymerase chain reaction

Given that this initial analysis showed higher rates of LBW in women receiving efavirenz-based regimens from early in pregnancy, we compared characteristics of women in this group with women taking other regimens. Women taking early HAART and receiving efavirenz had higher rates of TB compared with those on nevirapine and protease inhibitor-based therapy (28% [25/88] vs. 14% [25/183] and 10% [1/10] respectively, p = 0.01), lower median CD4 counts (138 cells/mm^3 ^vs. 155.5 cells/mm^3 ^vs. 164 cells/mm^3 ^respectively, p = 0.03), and longer median weeks on HAART (62.7 [IQR 33.1-86.4] vs.15.6 [IQR 10.7-25.8] and 17.1 [IQR 13.7-23.1] respectively, p < 0.001).

Overall, only 2% (26/1228) of all infants in the cohort were classified as very low birth weight (VLBW). More HAART-unexposed than exposed infants were VLBW (4% [10/224] vs. 2% [16/1004], p = 0.01). The mean CD4 cell count of the VLBW group did not differ from the remainder of the cohort (148 cells/mm^3 ^vs. 153 cells/mm^3^, p = 0.65). Rates of VLBW infants were similar in the early and late HAART groups and for all HAART regimens. Seven of the infants had known maternal risk factors for VLBW, including maternal history of previous miscarriage, previous preterm delivery, or hypertension. One infant in this group was HIV infected.

### Predictors of low birth weight

In univariate analysis evaluating associations between HAART and LBW, including both HIV-positive and HIV-negative infants, no significant associations were detected in the late HAART group (Table 3). However, in the early HAART group, receipt of an efavirenz-containing regimen, lower CD4 cell count, and maternal hypertension were associated with LBW. Although rates were low, neither smoking nor alcohol use was associated with LBW. In multivariate analysis, large effects of immunological status remained, with each 50 cells/mm^3 ^increase in CD4 cell count associated with a 57% reduction in the odds of LBW (95% CI 0.45-0.71, p < 0.001). There was a trend towards hypertension being associated with increased odds of LBW (AOR 2.1, 95% CI 0.92-4.82; p = 0.08). In multivariate analysis, the effect of efavirenz exposure was removed (AOR 1.02, 95% CI 0.46-2.25), and unexpectedly, nevirapine-based HAART was associated with a reduced odds of LBW compared with HAART-unexposed women (AOR 0.38, 95% CI 0.18-0.81, p = 0.01). Similar results were found in multivariate analysis of associations between HAART groups and LBW when modelling was restricted to only HIV-negative infants (data not shown).

**Table 3:** Multivariate logistic regression showing risk factors for LBW in HAART-exposed women irrespective of infant HIV status

Variable	Low Birth Weight (<2500 g)					
	
	Early HAART			Late HAART		
	
	Univariate analysis	Multivariate analysis (n = 341)		Univariate analysis	Multivariate analysis (n = 343)	
	
	OR (95% CI)	AOR (95% CI)	P	OR (95% CI)	AOR (95% CI)	P
**HAART-unexposed**	1.00	1.00		1.00	1.00	
**HAART-exposed**						
PI-based	0.58 (0.33-1.07)	0.45 (0.19-1.06)	0.068	0.58 (0.36-0.93)	0.52 (0.28-0.98)	0.04
NVP-based	0.58 (0.33-1.01)	0.38 (0.18-0.81)	0.01	0.76 (0.40-1.41)	0.70 (0.33-1.47)	0.34
EFV-based	1.86 (1.06-3.28)	1.02 (0.46-2.25)	0.96	0.73 (0.19-2.71)	0.51 (0.10-2.72)	0.43

**CD4 cell count**						
per category increase*	0.71 (0.60-0.83)	0.57 (0.45-0.71)	<0.001	0.94 (0.79-1.13)	0.91 (0.73-1.15)	0.45

**Maternal age**						
16-24 years	1.0	1.0		1.0	1.0	
25-29 years	1.24 (0.67-2.28)	0.80 (0.35-1.83)	0.60	0.96 (0.53-1.74)	0.98 (0.45-2.15)	0.96
30-34 years	1.45 (0.80-2.62)	1.19 (0.54-2.58)	0.67	1.26 (0.72-2.24)	0.81 (0.37-1.76)	0.60
≥35 years	2.02 (1.03-3.96)	1.71 (0.70-4.16)	0.24	1.40 (0.72-2.73)	1.62 (0.70-3.73)	0.26

**Hypertension**						
no	1.00	1.00		1.00	1.00	
yes	2.47 (1.18-5.17)	2.10 (0.92-4.82)	0.08	1.12 (0.48-2.60)	0.97 (0.40-2.36)	0.94

**Infant PCR**						
Negative	1.00	1.00		1.00	1.00	
Positive	2.54 (1.30-4.96)	3.28 (1.20-8.97)	0.020	1.93 (0.09-3.43)	2.37 (1.17-4.79)	0.02

HAART - highly active antiretroviral treatment; early HAART - HAART initiation <28 weeks of pregnancy; late HAART - HAART initiation at ≥28 weeks of pregnancy; PI - protease inhibitor, NVP - nevirapine; EFV - efavirenz; OR - odds ratio; AOR - adjusted odds ratio; CI (95%) - 95% confidence interval * CD4 cell categories (cells/mm^3^): 0-49 (baseline category), 50-99, 100-149, 150-199 and 200-250

### Infant outcomes: preterm birth

The overall rate of preterm birth in the cohort was 13.3% (145/1093). Rates were higher among women exposed to HAART during pregnancy than those unexposed (15% [138/946] vs. 5% [7/147], p = 0.002). In the analysis of early versus late HAART, infants whose mothers initiated treatment before 28 weeks had a higher rate of preterm birth compared with after 28 weeks (21% [81/389] vs. 5% [21/427], p < 0.001). HAART exposure was not associated with increased rate of extremely premature birth (6% [58/946] vs. 4% in unexposed women [6/147], p = 0.43).

### Predictors of preterm birth

In univariate analysis evaluating predictors of preterm birth in all infants (regardless of HIV status), in the early HAART group, every 50 cells/mm^3 ^rise in maternal CD4 cell count was associated with a 31% decrease in the odds of preterm birth (95% CI 0.58-0.83, p < 0.001, Table 4). This association remained significant in multivariate analysis (AOR 0.68, 95% CI 0.55-0.85, p = 0.001). In the multivariate analysis of specific HAART regimens, early exposure to any regimen was associated with preterm birth compared with HAART-unexposed infants, with receipt of early efavirenz and nevirapine associated with the higher odds of preterm birth (AOR 5.6, 95% CI 2.1-15.2, p = 0.001, and AOR 5.4, 95% CI 2.1-13.7, p < 0.001, respectively), than protease inhibitor-containing regimens (AOR 3.0, 95% CI 1.1-8.4, p = 0.04). Neither smoking nor alcohol use was associated with preterm birth in this analysis. Drug regimen was not associated with preterm birth in the late HAART group.

**Table 4:** Multivariate logistic regression showing risk factors for preterm birth in HAART-exposed women irrespective of infant HIV status

Variable	Preterm Birth (<37 weeks)					
	
	Early HAART			Late HAART		
	
	Univariate analysis	Multivariate analysis (n = 455)		Univariate analysis	Multivariate analysis (n = 477)	
	
	OR (95% CI)	AOR (95% CI)	P	OR (95% CI)	AOR (95% CI)	P
**HAART-unexposed**	1.00	1.00		1.00	1.00	
**HAART-exposed**						
PI-based	3.39 (1.38-8.36)	3.00 (1.07-8.38)	0.036	0.64 (0.23-1.76)	0.70 (0.23-2.13)	0.53
NVP-based	6.30 (2.72-13.56)	5.41 (2.14-13.70)	<0.001	2.10 (0.79-5.59)	1.88 (0.61-5.80)	0.27
EFV-based	6.40 (2.60-15.65)	5.64 (2.09-15.16)	0.001	1.00 (0.12-8.56)	1.47 (0.15-14.10)	0.74

**CD4 cell count**						
per category increase*	0.69 (0.58-0.83)	0.68 (0.55-0.85)	0.001	0.78 (0.57-1.07)	0.80 (0.55-1.15)	0.22

**Maternal age**						
16-24 years	1.00	1.00		1.00	1.00	
25-29 years	0.90 (0.42-1.89)	0.91 (0.37-2.21)	0.83	5.35 (1.2-23.75)	9.17 (1.17-72.0)	0.035
30-34 years	1.12 (0.54-2.33)	1.08 (0.45-2.57)	0.86	2.22 (0.46-10.66)	3.46 (0.41-29.45)	0.26
≥35 years	2.12 (0.98-4.57)	2.09 (0.83-5.25)	0.12	1.13 (0.16-8.18)	1.91 (0.17-21.66)	0.60

**Hypertension**						
no	1.00	1.00		1.00	1.00	
yes	1.83 (0.88-3.80)	1.95 (0.87-4.36)	0.11	0.96 (0.22-4.26)	0.84 (0.18-3.90)	0.83

HAART - highly active antiretroviral treatment; early HAART - HAART initiation <28 weeks of pregnancy; late HAART - HAART initiation at ≥28 weeks of pregnancy; PI - protease inhibitor, NVP - nevirapine; EFV - efavirenz;; OR - odds ratio; AOR - adjusted odds ratio; CI (95%) - 95% confidence interval

*CD4 categories: (cells/mm^3^): 0-49 (baseline category), 50-99, 100-149, 150-199 and 200-250

## Discussion

### HAART and low birth weight

The rate of LBW in this cohort (22%) was higher than the rate of 15% reported by UNICEF for all South African infants, regardless of HIV exposure [17]. Many of the factors associated with LBW in this study, such as CD4 count and hypertension, have been reported in previous studies [18,19]. Despite findings from a variety of other settings, in our cohort, lopinavir/ritonavir exposure was not associated with LBW in early or late HAART-exposed women compared with untreated women [5,20]. We detected an association between LBW and efavirenz, but this did not persist after controlling for stage of HIV infection and other potential confounding variables.

Unexpectedly, in the early HAART group, rates of LBW were lower in women on nevirapine-based HAART than in untreated women. Nevirapine has previously been associated with increased risk of LBW [12], as well as found to be neutral with respect to LBW [21]. These data are the first to suggest a decreased rate of LBW. This finding might be due to the fact that untreated advanced HIV infection (CD4 count <250 cells/mm^3^) confers increased risk for low birth weight, and initiation of treatment reduces this risk. There could also be site-specific reasons why non-nucleoside reverse transcriptase inhibitors (NNRTIs) and not PIs were significant in this analysis. Women who initiated NNRTIs tended to be from CMJH, which handled more complicated pregnancies [22,23].

HAART-unexposed women had a high rate of LBW (27%) despite a relatively low preterm birth rate (5%). This might be explained by potential selection biases (discussed later). Additionally, HAART-unexposed women likely had a high risk of intra-uterine growth retardation due to their untreated advanced HIV infection with resultant immunological dysfunction (median CD4 cell count 191 cells/mm^3^).

Only 26 study infants were VLBW. In the small group of VLBW infants identified, no association was detected with maternal immunological status. Small numbers of VLBW infants limit this analysis.

### HAART exposure and preterm birth

In this cohort of HIV-infected women with CD4 counts ≤250 cells/mm^3^, early HAART exposure was associated with an increased risk of preterm birth compared with untreated women. This is consistent with previous studies demonstrating an association with preterm birth and *in utero *HAART exposure [4,5,10,24]. A unique element of our study is the focus on African women with low CD4 counts who are initiated on HAART for their own health. Additionally, reported rates of smoking (3.7%) and alcohol use (3.9%) across all our groups were low, unlike many European and North American studies, where smoking prevalence has ranged from 7.5% to 55% [6,7,25,26].

The association between preterm birth and NNRTI exposure was larger than with PI exposure. Reasons for this may include bias related to site, as women exposed to NNRTIs were more likely to have attended the clinic at CMJH, which cares for women with more complicated pregnancies, increasing the likelihood for women to have multiple risk factors for preterm birth. Efavirenz-exposed women had more advanced HIV than women on other regimens, and early efavirenz-exposed women had been on HAART longer than other groups (62.7 weeks vs. 16 weeks); however, in multivariate analysis, there was only a small attenuation in the odds ratio (from 6.4 to 5.6), and residual confounding may be present, particularly given the lack of data on TB among women in the cohort.

The finding of an association with early PI exposure during pregnancy and preterm birth is similar to many previous studies. In these studies, PI-based regimens were often prescribed for women with more advanced HIV disease, making it difficult to disentangle the role of HAART regimen and stage of disease on infant outcome. In our study, all women had CD4 counts <250 cells/mm^3^_, _reducing the confounding caused by large differences in the degree of immunosuppression.

Additionally, the only PI used in our cohort was lopinavir/ritonavir as compared with previous studies evaluating unboosted PIs [4,10]. Our results are consistent with recent randomized data from Botswana, in which a median of 11.3 weeks of lopinavir/ritonavir exposure during pregnancy was associated with a significant risk for preterm birth compared with women on non-PI regimens (21.4% vs. 11.8%, p = 0.003) [27]. Specific type of PI and the use of ritonavir may be important determinants of the overall risk for preterm birth.

### Study limitations

Information was only collected from women who were willing to be included in the clinics' databases. Characteristics of women declining participation may differ from those giving consent, thereby introducing selection bias. Also, bias was potentially introduced by the selection strategy for the control group (HAART-unexposed women), as these women received less antenatal care and had poorer health-seeking behaviours than their HAART-exposed counterparts. These factors could, in part, account for the higher rate of LBW in the HAART-unexposed group.

Also, as the HAART-unexposed group were recruited postpartum, it is possible that a high mortality among infants born very preterm in this group partly explains the relatively low preterm birth rate (5%) noted in these women. Similarly, differences in how gestational age was assessed might have biased estimates of foetal age. HAART-unexposed women were more likely to have missed antenatal care, and have gestation determined by examination of infants at birth.

The study design also has inherent limitations as preterm delivery might have occurred before HAART could be initiated. This could conceivably have spuriously elevated the preterm birth rate among HAART-unexposed infants, who would have received "late HAART" had they remained *in utero*. Since more HAART-exposed than unexposed infants were born preterm, this factor is unlikely to have affected the study outcomes.

A considerable amount of data are missing for some study variables, mostly as the study was conducted within routine clinical settings and in two sites that collected different data. TB information was only available from CMJH women, and those exposed to early efavirenz-based HAART had the highest rates of TB as this regimen was specifically used with TB co-infection to minimize drug interactions. Because TB has been associated with intrauterine growth restriction and preterm birth [28,29], incomplete data on TB co-infection may confound our findings. Similarly, HIV viral load is not routinely collected in the South African public system and thus was unavailable. HIV viral load prior to delivery is a useful marker of adherence to HAART regimen, the pre-eminent risk factor for MTCT, and may have a role in predicting other poor infant outcomes. Additionally, maternal body mass index was unavailable and may be an important factor for LBW and preterm birth [12,22].

Since haemodilution during pregnancy is associated with spuriously low CD4 cell counts, women whose CD4 counts were measured postpartum (mainly HAART-unexposed women) were potentially more immunologically suppressed than the HAART-exposed group.

Differences in HAART prescribing protocols at CMJH and RMH could have affected the study outcomes. Women from CMJH tended to have more complicated pregnancies, for example, more frequent use of alcohol, higher rates of maternal hypertension or diabetes, and previous miscarriage. Baseline CD4 cell counts, however, were similar between the two hospitals. We were unable to include clinic site in the multivariate models due to its co-linearity with drug regimen.

It is hard to differentiate the effects of maternal HIV disease and that of HAART exposure on infant outcomes. Indeed, the improvement in immune status of the HAART-exposed women probably contributed to increasing infants' birth weights. However, improved maternal immune status would not explain the higher preterm birth rate in the HAART-exposed as compared with the unexposed women, which seems independent of HIV disease stage and might be due to HAART exposure or to the potential biases we have described. Finally, it is important to note that the findings of this study are likely to pertain only to women with marked immunological suppression, and may not extend to women with less advanced HIV disease.

## Conclusions

In this cohort of immunocompromised women from South African antenatal clinics, HAART exposure was not associated with LBW; the strongest predictor of LBW was lower maternal CD4 cell count. Early HAART exposure to any regimen and low maternal CD4 cell count were associated with increased rates of preterm birth. Of note, in this cohort, immunosuppression emerged as an important risk factor for both LBW and preterm birth, with every gain of 50 cells/mm^3 ^associated with considerably reduced risk of both adverse outcomes. This finding highlights the importance of early HIV diagnosis and staging during pregnancy to accelerate HAART initiation in women who qualify.

The recent South African guideline change to increase the CD4 count treatment threshold to 350 cells/mm^3 ^may help minimize adverse pregnancy outcomes related to immune compromise [30]. Further study is warranted to evaluate the impact of using even higher CD4 cell count thresholds, as this is a modifiable factor that could reduce morbidity and mortality for both mothers and newborns. Our findings contribute to the limited knowledge about antenatal HAART exposure in the African context and the methodological challenges of evaluating this topic. It is reassuring that the risks attributable to HAART appear relatively small, and are outweighed by the strong benefits for prevention of MTCT, as well as for maternal and infant morbidity and mortality.

## List of abbreviations used

AIDS: Acquired immunodeficiency syndrome; ANC-ARV: Antenatal and antiretroviral (clinic); AOR: Adjusted odds ratio; CMJH: Charlotte Maxeke Johannesburg Hospital; CI: Confidence interval; HAART: Highly active antiretroviral therapy (triple therapy); HIV: Human immunodeficiency virus; LBW: Low birth weight (less than 2.5 kg); MTCT: Mother to child transmission (of HIV); NNRTI: Non-nucleoside reverse transcriptase inhibitor; PCR: Polymerase chain reaction; PI: Protease inhibitor (lopinavir/ritonavir in this study); RMH: Rahima Moosa Mother and Child Hospital; SD: Standard deviation; TB: Tuberculosis; UNICEF: United Nations Children's fund; VLBW: Very low birth weight (less than 1.5 kg).

## Competing interests

The authors declare that they have no competing interests.

## Authors' contributions

KV conceived of the study. KV, AC, HR, MC and VB participated in study design. VB and KV collected data. MC performed the statistical analysis. MC, KV and RH analyzed data. KV, RH, VB, MC and HR wrote and edited the manuscript. All authors read and approved the final manuscript.

## Authors' information

KV: MBBCH (Wits), DCH (SA), Dip HIV Man (SA), MSc (Wits)

RH: MD, MPH

VB: BSc (Wits), MBBCh (Wits), DTM&H (SA), Dip HIV Man (SA)

MC: MBBCh (Wits) MSc (LSHTM) PhD (U.Gent) DFPH (UK)

AC: MB.ChB. (UNZA), DCH (SA), Dip HIV Man (SA) FCP (SA) Paed

HR: MBBChir (CANTAB MA (CANTAB) MRCGP (UK) DCH (SA) DRCOG (UK)
